# Neurodegenerative Disease–Associated Variants in TREM2 Destabilize the Apical Ligand-Binding Region of the Immunoglobulin Domain

**DOI:** 10.3389/fneur.2019.01252

**Published:** 2019-11-26

**Authors:** Hunter B. Dean, Erik D. Roberson, Yuhua Song

**Affiliations:** ^1^Department of Biomedical Engineering, University of Alabama at Birmingham, Birmingham, AL, United States; ^2^Department of Neurology, University of Alabama at Birmingham, Birmingham, AL, United States; ^3^Center for Neurodegeneration and Experimental Therapeutics, University of Alabama at Birmingham, Birmingham, AL, United States; ^4^Alzheimer's Disease Center, University of Alabama at Birmingham, Birmingham, AL, United States; ^5^Medical Scientist Training Program, University of Alabama at Birmingham, Birmingham, AL, United States

**Keywords:** Alzheimer's disease, frontotemporal dementia, neurodegenerative disease, triggering receptor expressed on myeloid cells 2, innate immune receptor, molecular dynamics

## Abstract

Single nucleotide variations in Triggering Receptor Expressed on Myeloid Cells 2 (TREM2) have been linked to both late-onset Alzheimer's disease and behavioral variant frontotemporal dementia (FTD), the latter presenting either in isolation or with cystic bone lesions in a condition called Nasu-Hakola disease. Models of the extracellular domain of TREM2 show that Nasu-Hakola disease–associated mutations are grossly inactivating by truncation, frameshift, or unfolding, that Alzheimer's disease (AD)–associated variants localize to a putative ligand-interacting region (PLIR) on the extracellular surface, and that FTD-associated variants are found in the hydrophobic core. However, while these disease-associated residues are predicted to play some role in disrupting ligand binding to the extracellular domain of TREM2, how they ultimately lead to disease remains unknown. Here, we used *in silico* molecular modeling to investigate all-atom models of TREM2 and characterize the effects on conformation and dynamical motion of AD-associated R47H and R62H as well as FTD-associated T96K, D86V, and T66M variants compared to the benign N68K variant and the common variant. Our model, which is based on a published 2.2 Å resolution crystal structure of the TREM2 extracellular domain, finds that both AD- and FTD-associated variants cause localized instability in three loops adjacent to the PLIR that correspond to the complementarity-determining regions (CDRs) of antibodies. This instability ultimately disrupts tethering between these CDRs and the core of the immunoglobulin domain, exposing a group of otherwise-buried, negatively charged residues. This instability and exposure of negatively charged residues is most severe following introduction of the T66M variant that has been described as causing FTD even in the heterozygous state and is less severe following introduction of variants that are less strongly tied to FTD or of those associated with AD. Thus, our results provide further evidence that the proposed loss-of-function caused by neurodegenerative disease–associated variants may be driven by altered conformational stability of the ligand-interacting CDR and, ultimately, loss of affinity or specificity for TREM2 ligands.

## Introduction

Triggering Receptor Expressed on Myeloid Cells 2 (TREM2) is an innate immune receptor found on myeloid-lineage immune cells, including dendritic cells, monocytes, and tissue-resident macrophages such as osteoclasts in bone and microglia in the brain (1). Heterozygous variants including R47H and R62H are risk factors for Alzheimer's disease (AD), while homozygous loss-of-function in TREM2 causes Nasu-Hakola disease, a severe, early-onset demyelinating dementia presenting as a frontotemporal dementia (FTD) syndrome with cystic bone lesions (2–13).

TREM2 variants have also been linked to FTD without bone involvement. This is most convincing for the T66M variant, as both homozygous (6, 11) and heterozygous (10, 12) carriers developing FTD have been described in families carrying this variant. Patients carrying T96K have been found in some studies to be at risk for FTD, particularly in African American cohorts (14); however, T96K is in linkage disequilibrium with the L211P and W191X variants (15) and it is not known whether the T96K variant drives the increased risk of FTD. Similarly, the D86V variant was first established in a pair of Turkish sisters that developed an FTD-like syndrome and carried compound heterozygous D86V and Y38C variants (7). However, while patients carrying homozygous Y38C have been reported to develop FTD (7), FTD patients with isolated heterozygous or homozygous D86V variants have not been reported, leaving questions about the pathogenicity of this variant. Notably, while evidence for the FTD syndrome without bone involvement was initially derived from familial studies, some studies have found population-level association between these variants and FTD in Belgian (9) and Italian (12) cohorts but not in other Western European cohorts (10, 16–18). Compared to the more well studied AD-associated variants, little is known about potential pathogenic mechanisms of these putatively FTD-associated variants.

**Figure 1:**
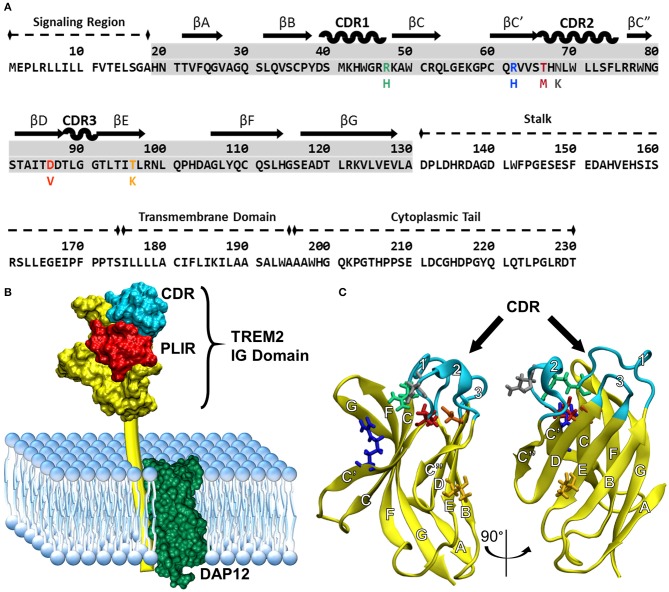
Common variant TREM2 3D-structure and amino acid sequence showing FTD- and AD-associated variants, functional domains, and secondary structure. **(A)** Sequence of TREM2 isoform 1 in humans with labeled domains. Secondary structure is mapped above the highlighted IG domain (gray) for reference. Colored amino acids represent the variants examined in this study with the mutated amino acid listed below the common. **(B)** Cartoon of TREM2 interaction with DAP12 in the cell membrane, including the complementarity-determining region (CDR) and putative ligand-interacting region (PLIR). The structure of the CV TREM2 immunoglobulin (IG) domain has been solved with X-ray crystallography, but the transmembrane and cytoplasmic domains have yet to be characterized. **(C)** Labeled structure of the TREM2 IG domain. Residues 21–129 from the 2.2 Å crystal structure of the TREM2 IG domain (PDB: 5ud7) are shown in yellow from two views with the CDR highlighted in cyan. Other residues of interest are shown for CV in licorice and colored to match the variants in panel A (green: R47, blue: R62, red: T66, gray: N68, orange: D86, yellow: T96). β-strands are labeled with letters and CDRs with numbers according to standard conventions for V-type immunoglobulins (19).

Understanding how these variants alter the structure of TREM2 is critical for understanding underlying disease mechanisms. The natively folded TREM2 protein is a 230-residue transmembrane receptor consisting of a signal peptide (residues 1–18), a V-set immunoglobulin (IG) domain (residues 19–130), a short connecting stalk (residues 131–174), a single-pass transmembrane region (residues 175–195) that associates with the adaptor protein DAP12 for signaling, and a C-terminal cytoplasmic tail (residues 196–230) (Figures 1A,B). Like other V-set immunoglobulins, the TREM2 IG domain forms a β-sandwich composed of nine antiparallel β-strands, which are lettered A–G including additional C' and C” strands according to the Williams and Barkley conventions (19) (Figure 1C).

The IG domain of TREM2 mediates its immune function. This domain can be shed through cleavage at the H157-S158 bond by ADAM10 or ADAM17, releasing a soluble form of TREM2 (20–23) that is still capable of mediating many of TREM2's immunomodulatory roles (24). TREM2 immune function is driven at least in part by the IG domain binding ligands that are associated with cell damage and death, including myelin debris (25), apoptotic neurons (26, 27), and extracellular plaques (28). The IG domain also binds other extracellular molecules associated with neurodegeneration including anionic lipids (29), apolipoproteins (including ApoA, ApoE, and CLU) either isolated or in lipoprotein particles (30–32), and amyloid-β (33–35). However, while the downstream effects of TREM2 ligand binding on cytokine production, phagocytosis, and other immune activities have been examined, the effects of changes in TREM2 structure on its ability to bind ligands remain mostly unknown. In particular, more detailed data regarding how neurodegenerative disease–associated variants in TREM2 affect its structure and dynamics, and in turn its ability to bind ligands, would enable development of more precise therapeutics to target TREM2 in neurodegenerative disease.

There are several gaps in understanding the structural effects of TREM2 variants on ligand-binding domains in the IG domain. The AD-associated R47H and R62H variants impair binding of specific AD-associated ligands (29–31, 33, 36–39), although the mechanism of this impaired binding is not fully clear. Nasu-Hakola disease–associated coding mutations tend to be grossly inactivating, usually by early truncations—such as E14X (3), Q33X (4), W44X (2), or W78X (2)—or frameshifts—such as G90VfsX99 or A105RfsX84 (5). In contrast, the FTD syndrome without bone involvement tends to arise when patients have some remaining TREM2 activity, including late truncations—such as W198X (8)—or in a variety of point mutations—including T66M (6), D86V (7), and T96K (10) (Figure 1A). In addition, FTD-associated variants have differential effects on binding for different ligands, suggesting that changes at the ligand binding site(s) may be more complex than simple unfolding in the region (37, 38, 40). Altogether, while the disease associations, whether in specific families or at the population level, have been documented for many of the known variants in TREM2, the structural rationale for how they associate with a variety of disease states remains poorly explored, particularly for putatively FTD-associated variants.

Much of the existing structural information about TREM2 is derived from two similar crystal structures of the IG domain of the common variant (CV) of TREM2 and one crystal structure of the AD-associated R47H variant of TREM2 (40–42). While most members of the IG superfamily bind ligands at or near a set of apical loops equivalent to the complementarity-determining regions (CDRs) of antibodies (43, 44), the crystal structures of TREM2 identify a prominent, positively charged patch of surface-exposed residues that is well conserved between species but not found in other members of the TREM family (40). Interestingly, while variants associated with AD are believed to primarily disrupt surface interactions at this putative ligand-interacting region (PLIR), variants associated with FTD occur primarily in the hydrophobic core where they are predicted to sterically disrupt packing of the IG domain (Figure 1C) (40). Notably, many of these residues sit in the region between the CDR and the PLIR, and thus may carry out their pathogenic role by destabilizing one or both of these regions to impair ligand binding (40, 42). In particular, an X-ray crystal structure of the R47H variant appears to show disruption of CDR2 as well as electrostatic changes in the nearby PLIR; however, the nature and extent of these changes are difficult to determine because of missing residues in the reported structure of the loop (42).

To help understand the molecular mechanisms underlying TREM2's involvement in FTD, we compared five disease-associated variants (spanning strongly to weakly disease-associated) and one benign variant to the CV using *in silico* molecular dynamics (MD) simulations. Specifically, we investigated the TREM2 IG domain containing the more convincingly FTD-associated T66M variant or the more tenuously FTD-associated T96K and D86V variants to determine their structural effects. To identify which structural effects were specific to FTD-associated variants or were more generally associated with neurodegenerative disease, we compared these three FTD-associated variants to the more common AD-associated R47H and R62H variants. Patients carrying one copy of the relatively rare R47H variant are consistently found to be at two to four times increased risk for developing AD (8, 9, 17, 45–48). In contrast, the more common R62H variant is only associated with a 40–70% increased risk (38, 45, 49), suggesting that any structural effects on TREM2 shared between the two AD-associated variants may be less severe in R62H than in R47H. N68K has been identified as a population variant (46) but has not yet been reported in patients with FTD or AD and has been found to have no detectible effect on TREM2 folding or aggregation (40), making it a useful comparison as a likely benign variant. These six variants thus represent a spectrum of strength of clinical evidence ranging from the most strongly FTD-associated (T66M), to weakly FTD-associated (T96K and D86V), to likely benign (N68K), with comparisons to AD-associated variants (R47H > R62H).

Examining these six variants in comparison to CV TREM2, we tested the structural hypothesis that variants in buried FTD-associated residues lead to TREM2 loss-of-function by disrupting stability of the PLIR or CDR. We provide evidence that the weakly FTD-associated T96K and D86V variants, as well as the AD-associated variants R47H and R62H, cause structural changes that are similar to those caused by the strongly FTD-associated T66M variant, although to a lesser degree. Our findings refine understanding of the impact of point mutations on the structural stability of TREM2 and give credence to a role for the apical CDR in neurodegenerative disease.

## Results

### Analysis of Equilibration

**Figure 2:**
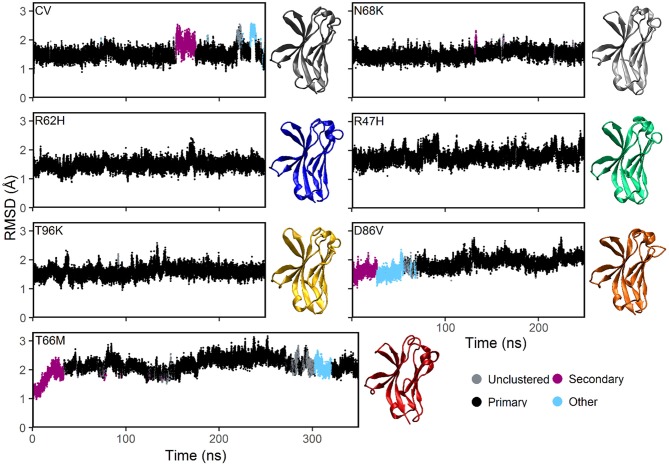
MD simulations of all examined isoforms of TREM2 equilibrate within 250–350 ns based on RMSD. Clustering analysis of RMSD profiles for the CV, FTD-, and AD-associated variants of TREM2 relative to the initial conformations. The starting conformation for each variant model was generated by mutating the residue of interest from the CV X-ray structure (PDBID: 5UD7). In the *dbscan* algorithm, clusters are ranked separately in each isoform by population. The first and second ranked clusters for each isoform are colored black and pink respectively to differentiate them from lower clusters (cyan) or unclustered frames (gray). CV, N68K, R62H, R47H, T96K, and D86V were simulated for 250 ns. T66M was run for an additional 100 ns to confirm stable equilibration. Representative structures (i.e., medoid of the most populated [black] cluster) are shown for each of the seven examined TREM2 IG domains. The same compact β-sandwich is observed in all seven TREM2 isoforms, but the conformations of the apical loops differ slightly between variants.

Plotting the root mean square deviation (RMSD) of all Cα atoms as a function of time for CV and N68K, R62H, R47H, D86V, and T96K variants of TREM2 over 250 ns revealed that the simulated systems reach equilibration after the first 100 ns of simulation (Figure 2). Although the RMSD of TREM2 containing the T66M variant failed to reach a single stable plateau over the initial 250 ns simulation, plotting the RMSD over a 350 ns simulation revealed a steady oscillation indicative of equilibrium. Based on RMSD analysis, the last 150 ns from the 250 ns trajectories of CV, N68K, R62H, R47H, D86V, and T96K, as well as the last 250 ns from the 350 ns trajectory of T66M, were used for further analysis.

To further analyze the degree of equilibration and to identify highly probable conformations for each isoform of TREM2, we applied the RMSD-based *dbscan* clustering algorithm (50) to the CV and disease-associated variant–containing TREM2 protein conformations isolated from the MD trajectory (Figure 2). Clustering with *dbscan* revealed a stable primary cluster (black) representing at least 100 ns for each isoform of TREM2. From the equilibrated region, the most populated cluster was identified for each isoform, and a representative medoid frame was chosen (Figure 2). While these medoids showed some small differences in secondary structure caused by the variants, particularly near the apical loops, all variants appeared to maintain the compact β-sandwich expected for the TREM2 IG domain. Together, these findings suggest that all of the isoforms of TREM2 that we examined equilibrate without any large RMSD changes that would indicate domain changes or gross misfolding.

### Fluctuation and Stability of the Overall Structure

**Figure 3:**
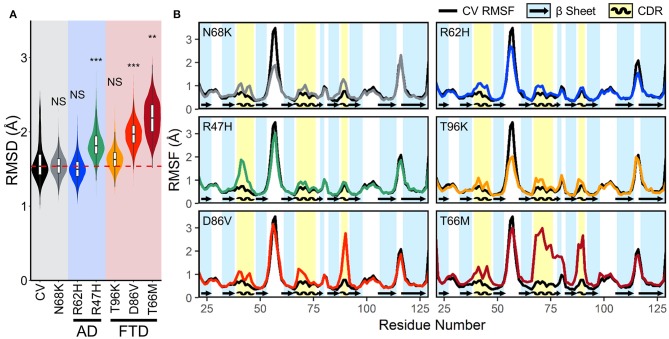
FTD- and AD-associated variants of TREM2 cause increased fluctuations in all three CDRs at equilibrium. **(A)** Orchestra plot showing distributions of the RMSD of the Cα atoms of each isoform of TREM2 averaged over the equilibrated trajectory, using the violin plot to show probability density and the contained box-and-whisker plot to show quartiles. The median of the CV protein is shown as the dotted red line for comparison. *P*-values are shown based on comparison to CV (NS, not significant, **P < 0.01, ***P < 0.001, Welch's ANOVA, Games-Howell *post-hoc*). **(B)** Comparison of root mean squared fluctuations (RMSFs) between CV TREM2 and TREM2 containing variants. RMSF is a measure of conformational flexibility of each residue averaged over the equilibrated trajectory. Each panel is overlaid on CV values (black lines) for comparison with reference β-strands (blue; arrows) and CDRs (yellow; waves) highlighted.

To quantify the conformational stability that occurs in each isoform of TREM2 at equilibrium, we plotted the distributions of the RMSD values from the equilibrated period. These orchestra plots revealed that proteins containing the R47H, D86V, and T66M variants, especially T66M, reached a greater distance from initial configuration than CV (Figure 3A). This conformational change, alongside the wider distribution in D86V and T66M variants, suggests that the equilibrated trajectories may include significant regional or domain fluctuations not present in the CV.

Root mean squared fluctuation (RMSF) measures conformational flexibility of each residue over the equilibrated simulation trajectories. It can be used to evaluate which regions of the protein contribute most to the conformational stability and motion of the protein at equilibrium. To elucidate whether broader changes in protein conformation revealed by RMSD resulted from changes in structural stability, we compared backbone RMSF of each variant to the CV (Figure 3B). Plots of RMSF revealed small increases in fluctuations of all three CDRs in models containing the N68K, R62H, and T96K variants, which were even more pronounced in CDR3 of models containing D86V and T66M variants and CDR1 of R47H. In addition, TREM2 containing the T66M variant showed a broad region of increased fluctuation at CDR2, including the entire βC” strand that immediately follows CDR2, suggesting that the T66M variant may disrupt some interaction that is important for tethering a large segment of this region. Outside the CDR, the βC-βC' loop—which can be seen as the most flexible peak in CV TREM2—showed a decrease in fluctuation in the N68K, R62H, and T96K variants, although the significance of this region is unknown. Altogether, these variant-induced increases in flexibility—in all three CDRs and the βC” strand—are most notable in the strongly FTD-associated T66M and strongly AD-associated R47H variants, and present to a lesser extent in the other examined variants. This pattern seems to provide initial evidence for T66M as the highest severity FTD variant, D86V as intermediate severity, and T96K as having little or no change from the benign variant N68K or CV. Together these findings suggest that decreased conformational stability near the apical CDRs caused by disease-associated variants of TREM2 may represent a shared mechanism for neurodegenerative effects of both AD- and FTD-associated variants.

### Secondary Structure of the TREM2 IG Domain

**Figure 4:**
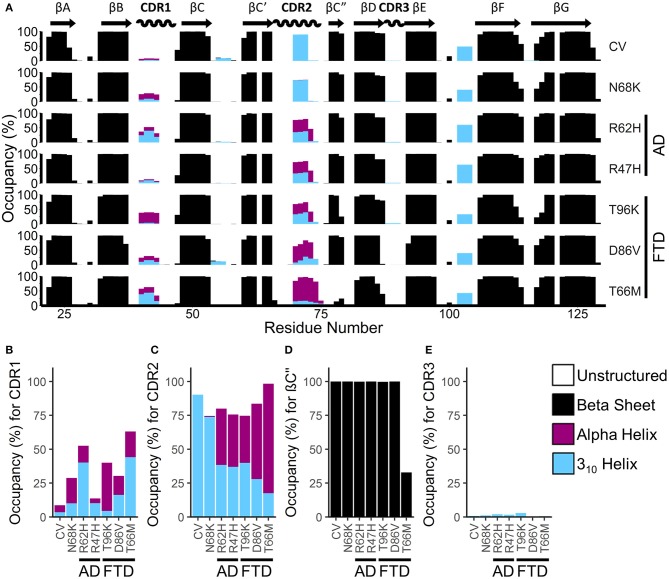
FTD- and AD-associated variants of TREM2 alter secondary structure in the apical portions of the CDR. **(A)** Percent occupancy by residue for three major classes of secondary structure for seven isoforms of TREM2. Percent occupancy in a secondary structure for each residue was assigned as percent of time that residue spends as β-sheet (black), α-helix (pink), or 3_10_ helix (cyan). **(B–E)** Total percent occupancy in the apical portions of CDR for three major classes of secondary structure for isoforms of TREM2. Note large (i.e., greater than 5%) increases in α-helicity caused by R62H, R47H, T96K, D86V, and T66M variants at **(B)** CDR 1 and **(C)** CDR2. **(D)** This increased α-helicity is associated with disruption of β-strand C” caused by the T66M variant. **(E)** However, there are no changes in CDR3 caused by any variant.

To identify gross changes in the secondary structure of TREM2 caused by FTD- or AD-associated variants, we used the *Define Secondary Structure of Proteins* (DSSP) algorithm (51) to determine the occupancy, or proportion of frames, that each residue spends as a β-strand, α-helix, 3_10_-helix, or unstructured strand, bend, or loop (Figure 4A). Consistent with the representative structures identified from RMSD analysis (Figure 2), analysis of secondary structure by DSSP revealed stable maintenance of the β-sandwich in all variants, with 8 of the 9 predicted strands showing >99% occupancy as a member of the β-sheet over the equilibrated trajectory. The exception was the region predicted to act as the βC” strand, which lost most β-strand structure in the T66M variant. On the other hand, secondary structure occupancy in CDR1 and CDR2 differed from CV in all disease-associated variants, while the benign N68K variant was the least different from CV.

To more closely examine the structural changes in these regions of interest, we plotted regional secondary structure—defined as the secondary structure that more than 50% of the residues in the region take during each frame over the equilibrated trajectories—as percent occupancy for the βC” strand and for all three CDRs (Figures 4B–E). Following the pattern noted in the residue occupancy, CDR1 was more helical with disease-associated variants than in CV, including in the benign N68K variant (Figure 4B). Similarly, CDR2 contained a short, 3–4 residue-long 3_10_-helix in most of the trajectories for CV and the benign N68K variant, which was partially replaced with the more stable 4+ residue-long α-helix in the disease-associated variants (Figure 4C). Accompanying this increased α-helicity in CDR2 of disease-associated variants, the βC” strand—which was present in >99% of CV, N68K, R62H, R47H, T96K, and D86V trajectories—was lost in TREM2 containing the T66M variant (Figure 4D). The loss of stability from the untethering of this β-strand is consistent with the increased flexibility noted in RMSF analysis of this region (Figure 3). Interestingly, CDR3, which also showed increased fluctuations in D86V and T66M variants by RMSF analysis, did not show large changes in secondary structure (Figure 4E), suggesting a different cause of its fluctuations, such as impaired tethering to the other CDRs. Together, these results suggest a similar pattern of severity in the FTD variants to that noted in RMSF analysis (T96K < D86V << T66M) as well as similar evidence of a shared mechanism between the FTD- and AD-associated variants.

### Motions of the CDR

**Figure 5:**
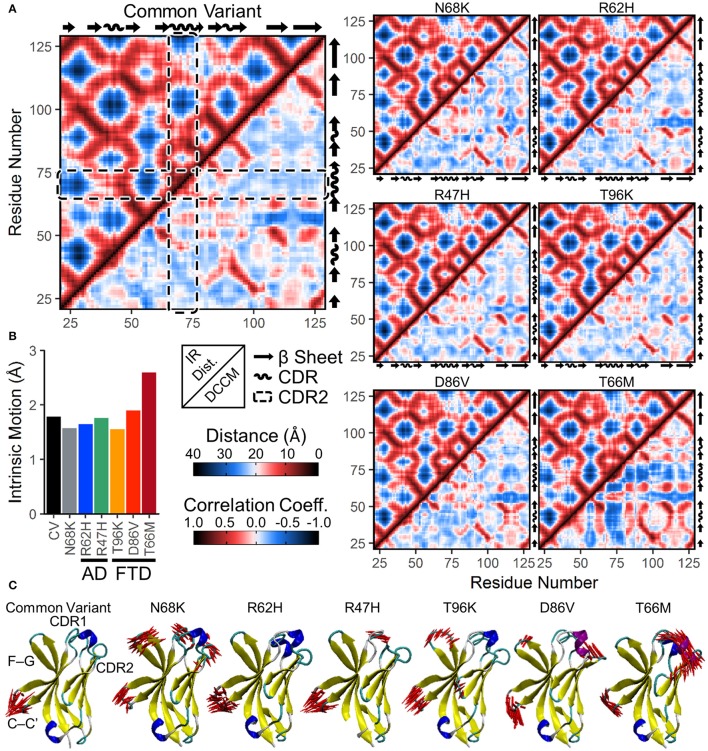
Increased anti-correlated motion from FTD- and AD-associated variants of TREM2 is driven by free movement of CDR2. **(A)** Inter-residue distance maps (top left) and dynamic cross-correlation maps (DCCMs; bottom right) for seven isoforms of TREM2. In the distance maps, nearby residues are shown in red and more distant residues in blue. In the DCCM maps, 1 represents perfectly correlated motion (darkest red) and −1 represents perfectly anti-correlated motion (darkest blue). CDR2 is outlined in the dashed boxes. While subtle differences in motion appear between variants, particularly near the CDR, the maps reveal no large domain movements. **(B,C)** Essential dynamics using principal component analysis in each isoform of TREM2. **(B)** Magnitude of intrinsic motion (i.e., motion not driven by translation or rotation of the protein) described by the first fifty eigenvectors of each TREM2 isoform. **(C)** Porcupine plots representing the contributory components of the first eigenvectors for each isoform of TREM2.

To examine whether changes in correlated motion of the CDR were associated with changes in the overall conformation of the protein, we generated a similar map of average distances between every pair of residues in the TREM2 IG domain (Figure 5A, upper left for each variant). Similar to the medoid representations (Figure 2) and the residue secondary structure analysis (Figure 4A), inter-residue distance maps for all six introduced variants were similar to CV (Figure 5A), suggesting no major domain movements or other large changes in conformation.

To determine whether protein conformational changes and the changes in regional secondary structure are associated with changes in particular dynamical motions, we built dynamic cross-correlation maps (DCCM) between all Cα atoms from the backbone of each isoform (Figure 5A, lower right for each variant). These maps, each of which is paired with the inter-residue distance map in the upper left to allow for easier comparisons of distance-dependent effects, show pairwise relative motion between each combination of residues. Similar to the results of RMSF (Figure 3) and secondary structure (Figure 4) analyses, T66M caused the most difference from CV, particularly in the increased anti-correlated motions (blue) between the CDR2 and the rest of the IG domain core (Figure 5A). In addition, both CDR1 and CDR2 showed mild increases in correlated motions (red) among nearby residues, consistent with the transition to a more structured helix noted in the secondary structure analysis.

In addition to DCCM analysis, essential dynamics (ED) analysis was performed using principal component analysis to decompose the trajectory into a series of eigenvectors that each describes a portion of the protein's total motion in space (52). We generated porcupine plots showing the largest contributions of the first three principal modes (Figure 5C). Consistent with the high degree of fluctuation near the CDR observed by residue RMSF (Figure 3), the first principal mode of each TREM2 isoform showed large amplitude perpendicular movements in the βC-βC' loop, with few additional contributory motions visible in CV TREM2 (Figure 5C). While these movements in the βC-βC' loop were still present in variant-containing TREM2 proteins, other motions become more prevalent in all but the R62H variant. These included similar motions of the βF-βG loops of proteins containing N68K, T96K, and D86V; motion of CDR1 in TREM2 containing N68K or R47H; and motion of CDR3 in TREM2 with N68K or the FTD-associated variants (Figure 5C). In addition, the large amplitude untethered fluctuations previously noted in CDR2 of TREM2 containing the T66M variant (Figure 3B) were also observed in the ED analysis of that variant (Figure 5C). Extending this analysis to the first three principal modes primarily revealed independent, statistically orthogonal motions in the same regions (Figure S1). However, the second principal mode of TREM2 with T66M was notable in that it appeared to be driven almost entirely by perpendicular motions of the βC” strand, likely tethering and untethering, suggesting that this motion is at least partly independent of the formation of the CDR2 α-helix.

The process of eigenvector decomposition in ED also generates a series of eigenvalues, each of which corresponds to, and describes the magnitude of, one principal component, with larger eigenvalues describing fluctuations on larger spatial scales. By convention, the first principal component from ED represents the direction of the largest conformational fluctuation (i.e., the largest eigenvalue) of the system during MD simulations, with successive components representing smaller and smaller contributions. Using this technique, we generated scree plots of the first 50 eigenvalues for all seven TREM2 isoforms, which each revealed loss of contributory information after the first five principal components (Figure S2). This quick drop in the magnitude of contributory motions suggests that most of the motions that differ between TREM2 isoforms are likely driven by a few large amplitude, low frequency oscillations. This was also supported by analysis of total intrinsic motion represented by the first 50 eigenvalues, which suggested that nearly all of the increased variance in RMSD distributions caused by variants in TREM2 (Figure 3A) can be explained by the increased total motion during the equilibrated period (Figure 5B). As before, these results point to T66M as being the highest severity variant with the other variants showing less or no deviation from CV.

### Electrostatic Potential of the PLIR

Non-uniform distribution of electrostatic potential over the surface of a solvated protein can contribute to the affinities of ligand binding and protein-protein interactions. To identify whether variants in TREM2 alter surface electrostatic potential, either by changes in the structure and motion of the region or by changes in the charge of the variant amino acids themselves, we used the *Adaptive Poisson-Boltzmann Solver* (APBS) to generate a map of electrostatic potential at the surface of the medoid conformations of each TREM2 isoform (53).

**Figure 6:**
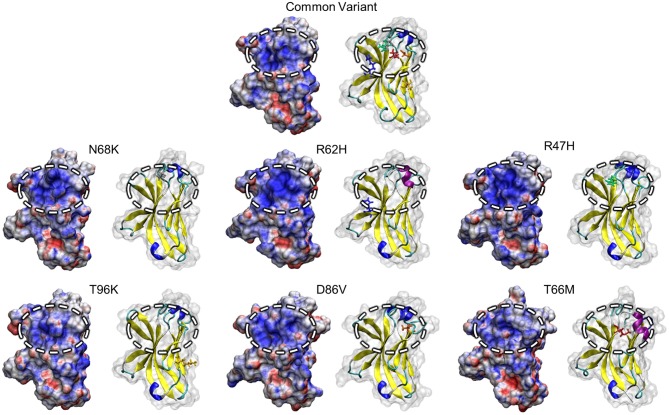
Positive electrostatic potential in the PLIR is maintained with FTD- and AD-associated variants of TREM2. Representative surface structures with mapped electrostatic potential for all examined isoform of TREM2 are shown alongside cartoons with mutated residues highlighted for reference. Electrostatic potential is used to define regions of positive (blue; >10 kT/e) and negative (red; <-10 kT/e) charge for each isoform. Surface electrostatics are shown for each isoform with the putative ligand-binding region represented by the circled upper-central region.

One region that has attracted attention as a potential driver of TREM2 loss-of-function, particularly in the AD-associated R47H and R62H variants, is a conserved patch of basic residues that has been proposed as a putative ligand-interacting region (PLIR, Figure 1B) for certain polyanionic ligands of TREM2 (40). Initial reports suggested that surface variants may cause neurodegenerative disease by disrupting the electrostatics of this PLIR—which includes residues of CDR2 and the βC” strand—thereby impairing binding of a subset of polyanionic lipids (40, 42, 54). Examination of the PLIR revealed small changes in surface electrostatic potential in the FTD-associated variants, but neither they nor the AD-associated variants exhibited the broad loss of positive electrostatic potential that has been predicted by previous studies (40, 42) (Figure 6). Together, these findings suggest that any effects on the PLIR caused by these variants are likely a function of the previously described changes in structural stability and not of additional changes in surface electrostatics in this region.

### Electrostatic Potential and Conformation of the CDR

We used the same approach to examine surface electrostatic potential over the CDR domains of each variant. Examination of the CDRs themselves did not reveal large differences in surface electrostatic potential caused by any of the four variants (Figure S3). However, in the medoid of TREM2 containing the T66M variant, the CDRs were further spread, exposing a small patch with negative electrostatic potential that could create a novel binding site (Figure S3). Notably, while this patch was not significantly exposed in the medoid conformations of other variant models, these models were built using only a single representative frame, and the because of the increased motion in this region in FTD-associated variants, there may be a subset of frames in the other variants where the patch is exposed.

**Figure 7:**
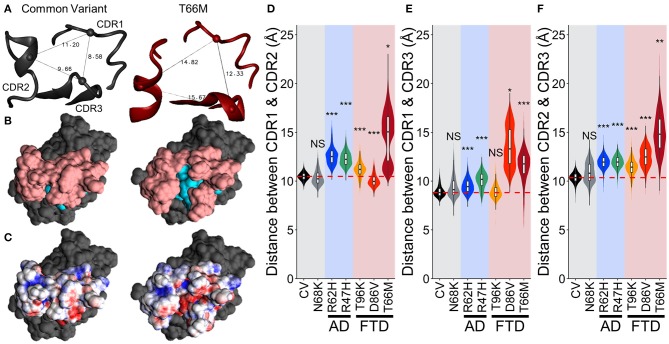
Increased fluctuations from FTD- and AD-associated variants of TREM2 separate the CDRs without altering TREM2 structure. **(A)** Representative structures showing distance measurements between alpha carbons of residues 45, 72, and 90—representing CDR1, CDR2, and CDR3 respectively—for examined isoforms of TREM2. **(B)** Representative surface structures from an apical view in the same orientation as **(A)**, indicating the CDR (pink) and novel binding site (cyan). **(C)** Electrostatic potential maps indicating regions of positive (blue) and negative (red) charge. **(D–F)** Orchestra plots showing distributions of distances between all three pairs of CDRs for each isoform of TREM2 over the equilibrated trajectory, using violins to show both normality and probability density and superimposed box/whiskers to show quartiles. The median of the CV protein is shown as the dotted red line in each for comparison. *P*-values are shown based on comparison to CV (NS, not significant, *P < 0.05, **P < 0.01, ***P < 0.001, Welch's ANOVA, Games-Howell *post-hoc*).

To examine whether this region with negative (red) electrostatic potential underlying the CDR was exposed more often with variants that disrupt stability in the region, we measured the distance between the peak alpha carbons of CDR1, CDR2, and CDR3 (residues 45, 72, and 90, respectively, representing the apical-most points of the CDR surface). The greater spread between these points in T66M (Figure 7A) was associated with exposure of residues normally buried in the CV (cyan in Figure 7B), creating the small patch of negative electrostatic potential (red in Figure 7C). We measured these inter-residue distances over time for each of the other variants to quantify the proportion of frames in which this novel CDR binding site may be exposed (Figures 7D–F). The distributions of inter-residue distances between each pair were consistent with the fluctuation and motion analyses, showing increased average distance and wider distributions caused by all five disease-associated variants. These results are once more consistent with a scale of severity between the disease-associated variants, with T66M having the greatest effect, D86V and R47H having intermediate effect, R62H having a smaller effect, and N68K and T96K having little or no effect on structural stability compared to CV TREM2.

## Discussion

In this study, we examined changes in the secondary structure, conformation, and dynamical motion of the TREM2 IG domain caused by the FTD-associated T66M variant, putatively FTD-associated D86V and T96K variants, and the putatively benign N68K variant, in comparison with AD-associated R47H and R62H variants. Our study revealed increased fluctuations (Figure 3), altered secondary structure (Figure 4), and increased motion and untethering (Figure 5) in a set of loops corresponding to the CDRs of variable chain antibodies in all five disease-associated variants. Interestingly, this did not result in notable loss of positive electrostatic potential directly at the PLIR (Figure 6), as previously predicted. Instead, increased motion of the CDR loops may be associated with exposure of a buried patch of negative electrostatic potential, which could alter ligand binding by the CDR domain (Figure 7). This pattern of variant-induced structural CDR instability is most notable in the better-documented FTD-associated T66M variant and to a lesser extent in the putative FTD-associated variants (D86V > T96K) and AD-associated variants (R47H > R62H).

Effects near the CDR are of particular interest as CDRs are crucial to the specificity of antibodies, and as such, loss of structure or stability in the CDR in particular may represent an important alteration in strength or specificity of ligand binding in TREM2. Altered structure of the CDR caused by increased motion in the disease-associated variants may also expose TREM2 to ligands that would not usually be able to bind, either inhibiting the ability of the physiological signals or causing pathological immune activation. The results of this study provide evidence for disrupted structural stability in FTD-associated variants of TREM2, particularly around the regions believed to be most associated with ligand binding.

It is interesting that this pattern of decreased stability near the CDR is also present in the AD-associated R47H and R62H variants, while the changes in electrostatic potential near the PLIR that have been predicted from crystal structures and binding studies did not manifest. This suggests that the changes in ligand binding previously noted for these variants may depend, at least in part, on binding at the CDR. Interestingly, untethering of the CDR2 loop was also recently noted in an X-ray crystal structure of the R47H variant extracellular domain (42), although the absence of this region in the published structure prevented further analysis of the effect. Notably, that same crystal structure was one of the early pieces of evidence for altered electrostatic potential in the PLIR in these variants; however, the same missing region around CDR2 may have contributed to that finding in the previous study, which is not seen in our simulations. Our simulations independently produce the finding of an untethered CDR region in the R47H variant and extend it to a variety of other disease-associated variants including another AD-associated variant, R62H, suggesting that it may be a general feature of neurodegenerative disease-associated variants in TREM2. This pattern is supported by examination of the reportedly benign N68K variant, which shows little deviation from CV in any of the examined measures of structural stability near the CDR.

Altogether, the degree of untethering at the CDR seems to correlate with disease susceptibility or severity between the different variants (N68K ~ T96K < R62H < R47H < D86V < < T66M) and provides additional support for a link between TREM2 D86V and FTD, with a mechanism similar to T66M, albeit less severe. However, our finding of weaker effects of T96K call into question whether its association with FTD is direct, or instead mediated by its linkage disequilibrium with the intracellular L211P and splice-variant–specific W191X variants. While experimental validation and further exploration into the exact role of the regions affected by these variants in ligand binding are recommended, this study provides a solid basis for further investigation into targeting the CDR with therapeutics as a possible treatment in certain cases of FTD or an enhancement to AD therapeutics that are currently being developed to target the PLIR. The variant structures investigated in this study provide a good starting point for examining the role of TREM2 structure in the binding of physiologic and pathologic ligands and may be directly useful in efforts toward rational drug design targeting TREM2 in other neurodegenerative diseases.

## Methods

### TREM2 IG Domain Structure

There are multiple crystal structures of the TREM2 IG domain in the protein data bank (41, 42). We chose the highest resolution 2.2Å crystal structure of the human TREM2 ectodomain expressed in HEK-293S cells (PDBID: 5ud7; Figure 1C) as our model of the CV human IG domain (42). The variant protein structures for R47H, R62H, T66M, N68K, D86V, and T96K were obtained based on crystal structure of CV human TREM2 obtained from the RCSB Protein Database (PDBID: 5ud7). For each examined variant, we mutated the amino acid in question using the *tleap* program included in AMBER14 (55). The obtained structures of CV and each variant were then energy minimized by steepest descent followed by conjugate gradient in AMBER14 to obtain initial structures for use in MD simulations.

### Molecular Dynamics Simulations

The secondary structure, conformational space and dynamical motion of each FTD- or AD-associated variant of TREM2 was explored by means of MD simulations. MD simulations for CV TREM2 and TREM2 containing variants in the IG domain were performed with AMBER14 (55) using the ff14SB force field (56). The MD simulations were performed in a periodic box with 2 nm of solvent between the protein edge and the box boundary to reduce periodicity artifacts. The periodic box was filled with TIP3P water and 150 mM NaCl added at random positions to approximate physiologic conditions. Additional Cl^−^ ions were added to each system at random positions to neutralize the protein charge. We first performed steepest descent minimization of the solvent water with the protein and ions restrained. This was followed by equilibration of the minimized water molecules with the protein and ions restrained at constant number-pressure-temperature at 50 K and 1 bar for 20 ps. The system was heated via a series of 10 ps constant number-volume-temperature MD simulations at 50, 100, 150, 200, 250, and 300 K. MD production simulations of 250 ns or 350 ns at number-pressure-temperature of 300 K and 1 bar were performed for all seven isoforms of TREM2. For all MD simulations, SHAKE constraints with relative tolerance of 1 × 10^−5^ were used on all hydrogen-heavy atom bonds to permit time steps of 2 fs. Electrostatic interactions were calculated by the particle-mesh Ewald method. The Lennard-Jones cutoffs were set at 1.0 nm.

### System Equilibration

To determine the degree of equilibration in our simulated systems, RMSD was calculated over the trajectory of each simulation. To compliment visual examination of RMSD, clustering analysis was performed based on the measured RMSD. Clustering analysis is useful to help resolve trajectories further by partitioning frames into groups with similar structural features, which may not be visible by RMSD analysis alone. The *dbscan* (density-based spatial clustering of applications with noise) algorithm was used as the clustering algorithm (50). In *dbscan*, points are considered as part of a single cluster if there is at least *n* other points within a neighborhood radius ε. To minimize bias, distribution plots of distance from the 5th–nearest neighbors (i.e., *n* = 5) were generated to parameterize the *dbscan* clustering algorithm for CV and disease-associated variants of TREM2, and the 5th percentile of distance in the resulting plot was selected for each as the neighborhood radius for that variant. The *dbscan* algorithm also generates an average structure for the population of each cluster called a medoid, which can be used to determine a representative conformation for use in further analysis. Based on these analyses, we determined the initial simulation time needed for each simulation to reach system equilibration.

### Conformational Flexibility

To characterize the differences in conformational flexibility caused by variants in TREM2, we calculated RMSF of individual alpha carbons for each residue along the TREM2 backbone over the equilibrated simulation trajectory. RMSF for each TREM2 that contained one of the four variants was compared with that for CV TREM2. To determine the overall protein conformational stability changes caused by TREM2 variants, the mean and standard error of RMSD of TREM2 CV and disease-associated variants over the equilibrated MD trajectories were calculated for the comparison.

### Secondary Structure

To characterize the effects of FTD- and AD-associated variants on TREM2 secondary structure based on the equilibrated MD simulation trajectories, we examined changes in secondary structure using the DSSP method in the *cpptraj* program of AMBER14 (51). This method assigns secondary structure based on calculation of the ideal (i.e., assumed to be 1.000 Å from the backbone N in the opposite direction from the backbone C = O bond) hydrogen bond energy with all nearby atoms. From these energies, the best two H-bonds for each atom are then used to assign the most likely class of secondary structure for each residue in the protein by comparing these energies to those of all secondary structures for protein models stored in the RCSB Protein Database. These calculations are performed for each equilibrated frame over the MD simulation and presented as a percent of time that each residue or any residue within a region of interest occupies a particular secondary structure.

### Dynamical Motion

To analyze the degree of correlated motions between residues for the studied TREM2 variants over the equilibrated MD simulation trajectories, we generated a DCCM for each TREM2 variant as well as the CV protein. Using a trajectory containing *M* frames of *N* residues, the DCCM is constructed as an N × N correlation matrix, *D*:

(1) $$D_{ij}=\frac{M^{-1}\sum_{t=1}^{M}\left[x_{i}\left(t\right)-\langle x_{i}\rangle \right]\left[x_{j}\left(t\right)-\langle x_{j}\rangle \right]}{\sqrt{M^{-1}{\sum_{t=1}^{M}\left\|x_{i}\left(t\right)-\langle x_{i}\rangle \right\|}^{2}}\sqrt{M^{-1}{\sum_{t=1}^{M}\left\|x_{j}\left(t\right)-\langle x_{j}\rangle \right\|}^{2}}}$$

Each correlation *D*_*ij*_ can have a value ranging from −1 to 1, where −1 represents perfectly anticorrelated motion, 1 represents perfectly correlated motion, and 0 represents perfectly independent motion between two residues *i* and *j*.

To determine which components of movement contribute most to the total motion at equilibrium, we separated the equilibrated motions into principal components using ED (52, 57). Similar to DCCM analysis, the ED algorithm constructs a matrix using residue displacements to represent motions that tend to occur together. However, where DCCM directly constructs an N × N correlation matrix using ensemble averages of vector displacements, ED first generates a 3N × 3N covariance matrix, *C*, of Cartesian displacements, *C*_*ij*_:

(2) $$C_{ij}=M^{-1}\sum_{t=1}^{M}[x_{i}\left(t\right)-\langle x_{i}\rangle ]\left[x_{j}\left(t\right)-\langle x_{j}\rangle \right]$$

This new matrix of normal vector displacements is then diagonalized and systematically filtered to generate a series of orthogonal eigenvectors, *V*:

(3) $$\lambda =V^{T}CV$$

Each eigenvector, along with its corresponding eigenvalue, λ, characterizes a principal mode of the total motion of the protein, where V describes the direction of motion and λ describes its magnitude. The eigenvalues can also be used to determine the contribution of each mode to the total motion of the protein according to the expression $\frac{\lambda_{i}}{\sum_{i}\lambda_{i}}$ or to measure the total intrinsic motion of the analyzed protein according to the expression $\sqrt{\underset{i}{\sum }\lambda_{i}}$. This allows ED to analyze not only whether the two particles move in the same or opposite directions as in DCCM, but also to extract more complex motions such as mutually perpendicular or rotational movements and describe their separate contributions to the total protein motion.

### Electrostatic Potential

To determine whether individual amino acid mutations cause changes in surface electrostatics, we generated electrostatic potential maps for each isoform of TREM2 using APBS (53). In brief, a representative conformation from the most populated cluster from dbscan was selected as our model. We then used the PDB2PQR software package (58) to generate AMBER radii and charges for each atom in the model and used these parameters along with the representative spatial conformation (i.e., the medoid of the most populated *dbscan* cluster) to generate a representation of the electrostatic potential at the surface of each isoform of TREM2. These models were then used as inputs to APBS to generate continuum maps of the surface electrostatic potentials for each isoform of TREM2.

### Regional Geometry

To identify the presence of large domain motions in the IG domain, we generated a map of pairwise distances between residues. To exclude the effects of residue rotation from this analysis, measurements were taken between the centers of mass of the C_α_'s for each pair of residues. Distances were measured for each pair at every frame of the equilibrated trajectory, and the averaged distances are presented as an N × N distance matrix to allow comparison with DCCM plots.

To examine the changes of the CDR shape caused by TREM2 variants, we calculated the mean and standard error of the inter-residue distances between the isolated residues 45, 72, and 90—which represent the most apical points of CDR1, CDR2, and CDR3, respectively—over the equilibrated MD trajectories for all seven TREM2 isoforms. Results for TREM2 CV and disease-associated variants were shown as Orchestra Plots for comparison.

### Statistical Analysis

Because of the frequent resampling of an isolated system used by MD simulations, adjacent frames within the trajectories often have high degree of autocorrelation. For this reason, the decorrelated values of observables (i.e., RMSD and inter-residue distance) from these trajectories were obtained as previously published by our lab (59, 60) based on the analysis of Chen and Pappu (61) to generate the independent values needed for meaningful statistical analyses.

To determine the autocorrelation time, τ, required for successive values, *x(t*_*i*_*)*, of an observable with mean value <*x*> to be statistically independent, we first calculated the autocorrelation coefficient, *A(*τ*)*:

(4) $$A\left(\tau \right)=\frac{m}{m-l_{\tau }}\frac{\sum_{j=1}^{m-l_{\tau }}\left(x\left(t_{j}\right)-\langle x\rangle \right)\left(x\left(t_{j+l_{\tau }}\right)-\langle x\rangle \right)}{\sum_{i=1}^{M}{\left(x\left(t_{i}\right)-\langle x\rangle \right)}^{2}}$$

For every possible *m* frames *{t*_1_*, t*_2_*,…t*_*m*_*}*, such that *1* ≤ *m* ≤ *M (*where *M* is the total equilibrated frames of the trajectory), with equal spacing Δ*t* = *t*_1+1_ – *t*_*i*_ and lag *l*_τ_ such that τ = *l*_τ_Δ*t*. Based on these correlation coefficients, a characteristic correlation time τ_*A*_ was calculated as the shortest τ for which *A(*τ*)* < *e*^−1^. Final decorrelated values of the observable were obtained by randomly isolating *M*Δ*t/2*τ_*A*_ separate blocks of *2*τ_*A*_ adjacent frames each from the total equilibrated frames, *M*, of the trajectory and averaging each resampled block into a single value. The entire subsample with *M*Δ*t/2*τ_*A*_ (rounded to the nearest integer) decorrelated values was used to calculate mean and standard error for each observable. The means, standard errors, and sizes of these subsamples were also used to compare between isoforms using Welch's ANOVA with a 95% confidence interval followed by Games-Howell *post-hoc* analysis for comparison of each variant to CV.

### Technical Specifications

MD simulations were performed on the University of Alabama at Birmingham's Cheaha Supercluster using 32 conventional 2.5 GHz Intel Xeon E5 series cores in parallel with OpenMPI v1.10.2. RMSD, *dbscan* clustering, DSSP, RMSF, ED, DCCM, and inter-residue distance analyses were carried out using the *cpptraj* program of AMBER 14.13 (55). Electrostatic potential was calculated using APBS 1.4.2.1 (62) and PDB2PQR 2.1.1 (58) software packages. Protein conformations, electrostatic potentials and PCA visualizations are presented using VMD 1.9.3 (63). The *NMWiz* plugin for VMD was used to generate porcupine plots for PCA (64). All other analyses were performed using R 3.5.2 (65) and all other plots constructed with the *ggplot2* package (66) in R.

## Data Availability Statement

All input command, coordinate, and topology files can be found in the GitHub repository (https://github.com/HBDean/FrontiersPaper2019). All molecular dynamics trajectories are stored in the Open Science Framework repository (https://osf.io/a6yqv/). All other relevant data for this study are included within the manuscript and its Supplementary Files.

## Author Contributions

HD, ER, and YS: conceived and designed the experiments, wrote, and edited the paper and acquired funding for the experiments. HD: performed the experiments and analyzed the data. YS: provided software and analysis tools, and guided computational study.

### Conflict of Interest

ER is an inventor on patents and patent applications related to the FTD-associated protein tau. The remaining authors declare that the research was conducted in the absence of any commercial or financial relationships that could be construed as a potential conflict of interest.
