# Supplementary material for: Neurodegenerative Disease–Associated Variants in TREM2 Destabilize the Apical Ligand-Binding Region of the Immunoglobulin Domain
Source: Front Neurol. 2019 Nov 26;10:1252. doi: 10.3389/fneur.2019.01252 (PMC6985895; doi:10.3389/fneur.2019.01252)
Supplement: Supplementary file 1 [file Table_1.DOCX]

**S1 Fig. Porcupine plots of the first three dominant PCA motions for isoforms of TREM2.** (A – C) Porcupine plots representing the contributory components of the (A) first, (B) second, and (C) third eigenvectors for each isoform of TREM2.

**
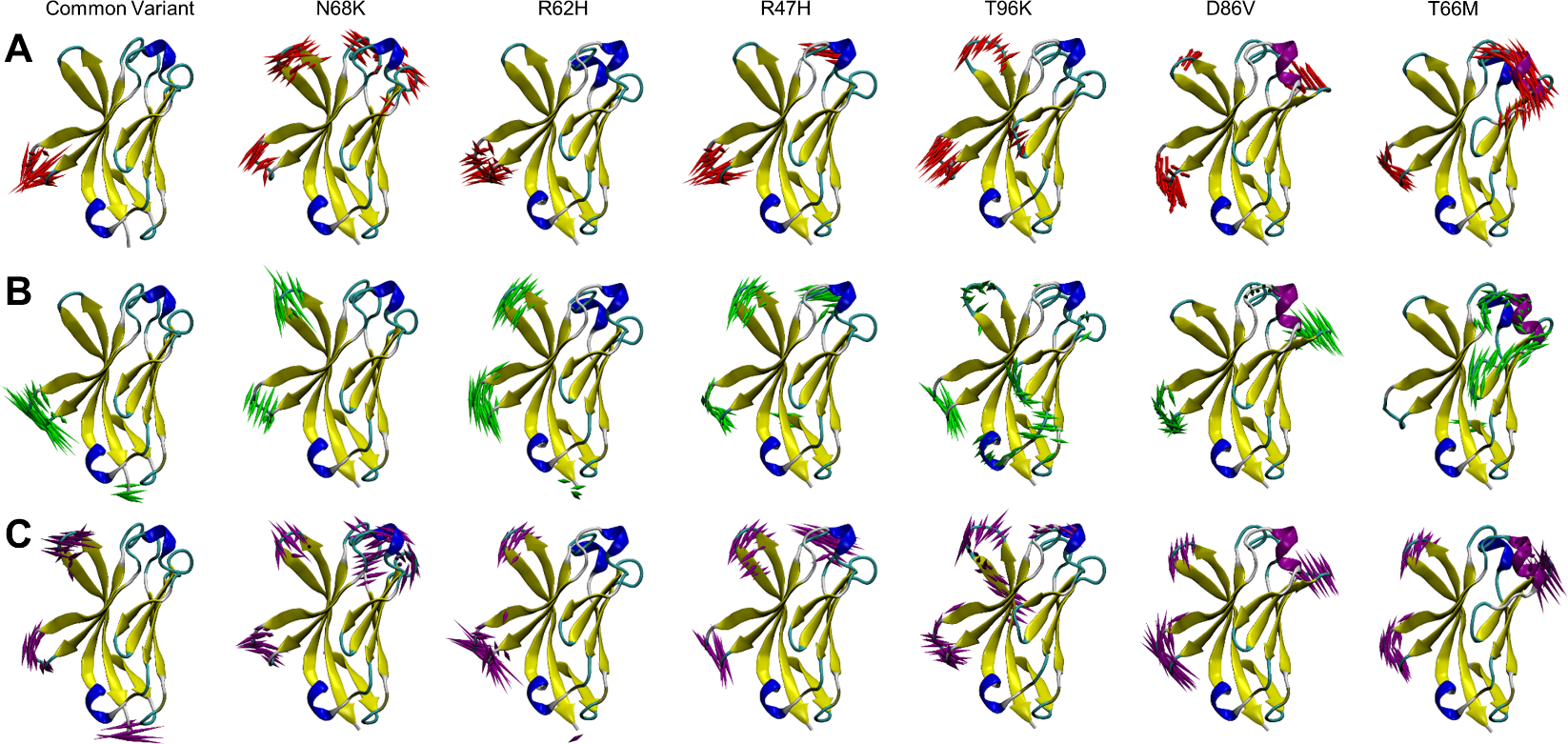
**

**S2 Fig. The first fifty eigenvalues for each variant as percent of total motion explained.** (A) Scree plots of the first fifty eigenvalues for each isoform of TREM2 as percent of total motion. (B) Overlay of cumulative variance from the data in (A).

**
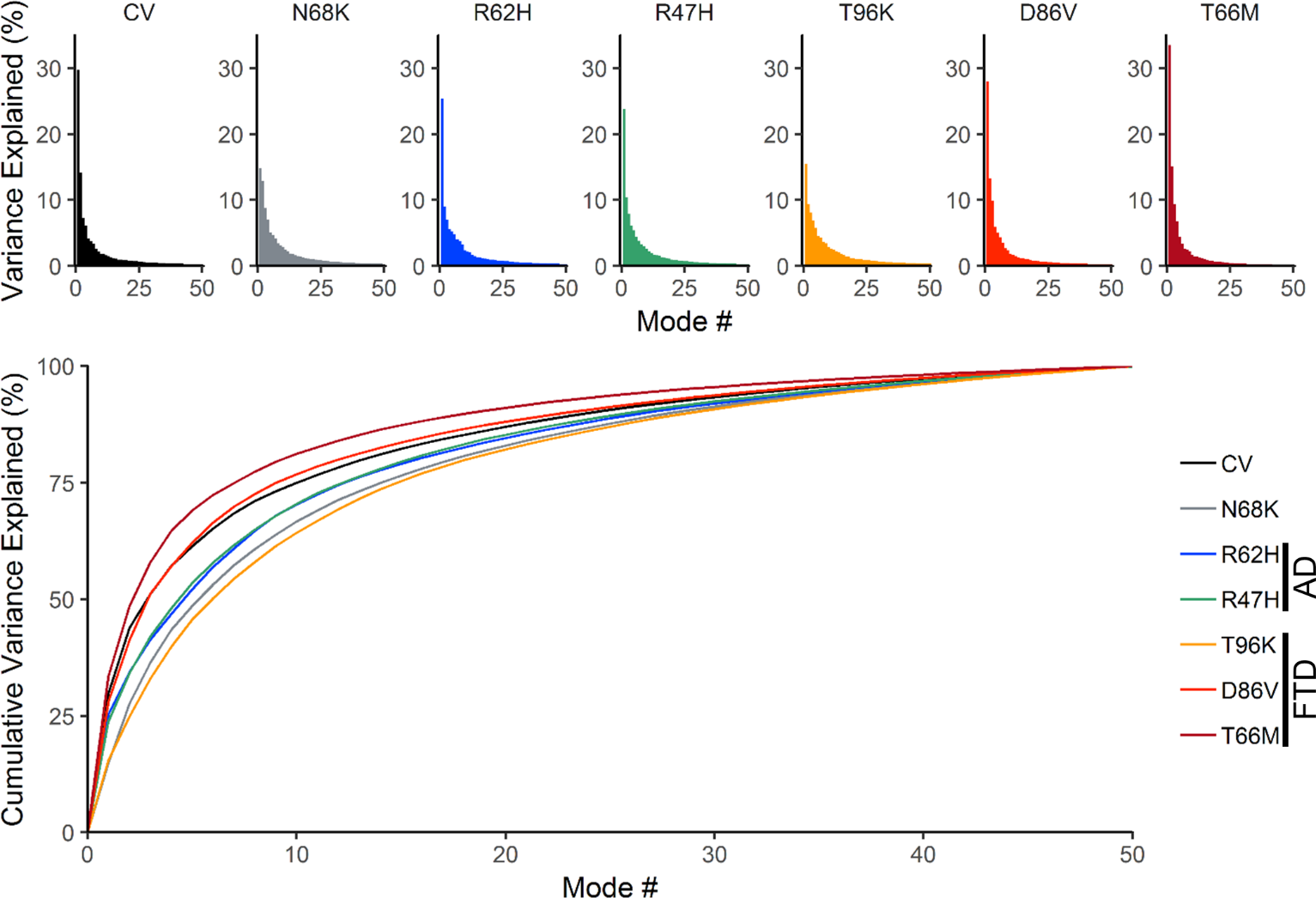
**

**S3 Fig. Electrostatic potential in the CDR is not disrupted by FTD-associated variants of TREM2.** Representative surface structures (left) alongside electrostatic potential maps (middle) and cartoons (right) for each examined isoform of TREM2. Surface structures highlight the CDR (pink) and novel binding site (cyan). Electrostatic potential maps indicate regions of positive (blue; >10 kT/e) and negative (red, <–10 kT/e) electrostatic potential. Note the region of negative electrostatic potential under the CDR that is exposed when loops are separated by the T66M variant.

**
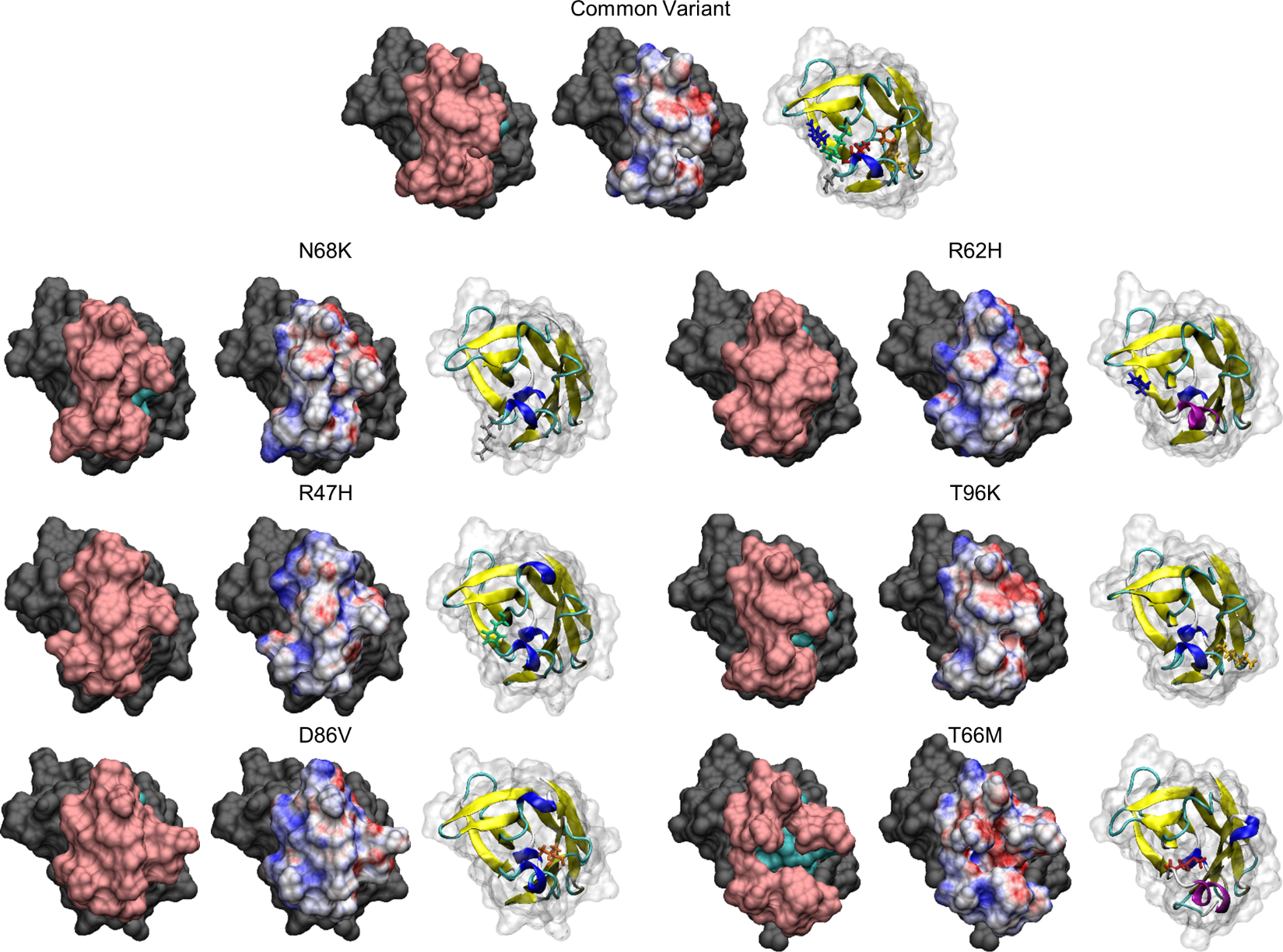
**
